# A Systematic Review and Meta-Analysis of the Effectiveness of Neuroprotectants for Paclitaxel-Induced Peripheral Neuropathy

**DOI:** 10.3389/fonc.2021.763229

**Published:** 2022-01-05

**Authors:** Alisha Joan Leen, Dominic Wei Ting Yap, Chong Boon Teo, Benjamin Kye Jyn Tan, Alex Molassiotis, Hiroshi Ishiguro, Sarah Wei Xian Fan, Raghav Sundar, Yu Yang Soon, Aishwarya Bandla

**Affiliations:** ^1^ School of Medicine, National University of Ireland (NUI) Galway, Galway, Ireland; ^2^ Department of Haematology-Oncology, National University Health System, Singapore, Singapore; ^3^ The N.1 Institute for Health, National University of Singapore, Singapore, Singapore; ^4^ Yong Loo Lin School of Medicine, National University of Singapore, Singapore, Singapore; ^5^ School of Nursing, The Hong Kong Polytechnic University, Hung Hom, Hong Kong, Hong Kong SAR, China; ^6^ Department of Medical Oncology, International University of Health and Welfare Narita Hospital, Chiba, Japan; ^7^ National University Cancer Institute, National University Health System, Singapore, Singapore; ^8^ Department of Radiation Oncology, National University Health System, Singapore, Singapore

**Keywords:** chemotherapy-induced peripheral neuropathy (CIPN), taxane, prevention, neuroprotection, paclitaxel-induced peripheral neuropathy, non-invasive

## Abstract

**Background:**

Paclitaxel-induced peripheral neuropathy (PIPN) is a disabling side effect of paclitaxel with few effective preventive strategies. We aim to determine the efficacy of pharmacological and non-pharmacological neuroprotective interventions in preventing PIPN incidence.

**Methods:**

Biomedical literature databases were searched from years 2000 to 2021 for trials comparing neuroprotective interventions and control. Meta-analysis was performed using the random-effects model. The primary outcome was the incidence of PIPN.

**Results:**

Of 24 relevant controlled trials, 14 were eligible for meta-analysis. Pooled results from seven non-pharmacological trials were associated with a statistically significant 48% relative reduction of PIPN risk with low heterogeneity. Conversely, pooled results from six pharmacological trials were associated with a significant 20% relative reduction of PIPN risk with moderate heterogeneity. Both pharmacological and non-pharmacological approaches appear effective in reducing PIPN incidence in the treatment arm compared to control (pooled RR < 1).

**Conclusion:**

Current evidence suggests that both interventions may reduce PIPN risk. Non-pharmacological interventions appear more effective than pharmacological interventions.

## Introduction

Chemotherapy-induced peripheral neuropathy (CIPN), a severe dose-dependent toxicity, often results in chemotherapy dose reduction or cessation, adversely affecting efficacy of chemotherapy itself. The resulting neurotoxicity profoundly impacts the quality of life of survivors. With increasing survival rates of cancer patients, there has been an emerging research interest in addressing the detrimental dose-limiting and long-term effects of CIPN (1, 2).

Paclitaxel, a taxane, is used to treat various cancers, including ovarian, breast, and lung carcinomas. Paclitaxel-induced peripheral neuropathy (PIPN) presents as predominantly sensory peripheral neuropathy, while motor and autonomic neuropathy occur to a lesser extent. A recent study showed that up to 80% of patients had neuropathic symptoms for up to 2 years post-treatment, with approximately 25% reporting severe symptoms of numbness and/or discomfort in their hands and feet (3, 4). Existing treatment is limited to dose management and symptomatic cure i.e. pain killers (1). Prevention of these toxic neuropathies impels clinical impact by delivery of the appropriate chemotherapy dose and improved quality of life.

Neuroprotective strategies are being explored to address this unmet medical need. Several interventions have been reported for preventing PIPN. Despite extensive research efforts and successfully reported preclinical studies, there is paucity of high-quality, consistent evidences of clinical successes (2). Trial designs vary in several parameters. Moreover, the absence of a standardized CIPN assessment approach contributes to the lack of clarity of outcomes reported (3).

Prevention of PIPN is crucial to ensure effective delivery, hence maximal benefit, of paclitaxel. PIPN is often irreversible and deteriorates the quality of life of affected patients financially (unemployment and medications), psychologically, and physically, leading to economic and healthcare costs (5). Interventions reducing incidence of CIPN could be key adjuncts to future oncological treatment using such neurotoxic chemotherapeutic agents.

The objective of this systematic review and meta-analysis was to determine the effectiveness of pharmacological and non-pharmacological interventions in reducing the incidence of PIPN. This meta-analysis is designed to better assess the potential efficacy of such neuroprotective interventions currently tested in humans and provides a better understanding of the current state of neuroprotectants. With more interventions (pharmacological and non-pharmacological) gaining recent focus towards the prevention of this disabling side effect, this systematic review aims to consolidate available evidence to help identify promising directions and address factors that would propel development of more wholesome future clinical trials for PIPN prevention.

## Methods

### Inclusion Criteria

Only controlled trials were included—randomized controlled trials (RCTs) and internal controls (to accommodate non-pharmacological studies). Studies with adult participants, of either sex and diagnosed with any cancer or stage, and scheduled to undergo paclitaxel chemotherapy were included. The intervention arm would include pharmacological or non-pharmacological interventions. The control arm comprised participants assigned to the placebo or control arm. Self-controlled studies were also included, where an intervention was administered unilaterally/one limb and the PIPN burden compared between the two limbs, e.g., frozen gloves applied to one hand per participant. The primary outcome was incidence of PIPN. Studies that investigated symptomatic treatment of pre-existing peripheral neuropathy were excluded. i.e., only studies that excluded patients with pre-existing grade 1 neuropathy were accepted. Studies that investigated combinations of antineoplastic drugs (which included paclitaxel) were excluded.

### Search Method

We searched MEDLINE (*via* PubMed), Embase, and Scopus from January 2000 to Sept 2021. The search strategy included the medical subject headings “paclitaxel neuropathy” with “clinical” and “oncology” filter. The full search string may be found in **Supplementary Table 1**. The results were hand-searched for eligible studies, and their reference lists were searched for any other relevant studies.

### Data Collection and Analysis

#### Selection of Studies

All titles and abstracts retrieved by electronic search were downloaded to a reference management database, duplicates were removed, and the remaining references were examined independently by two review authors (AL and DY). Studies that failed to meet the eligibility criteria were excluded and copies of the full texts of potentially relevant references were obtained. The eligibility of the retrieved papers was assessed independently, and disagreements were resolved by discussion between the two authors.

#### Data Extraction and Management

Two reviewers (AL and DY) independently extracted the data on characteristics of patients (inclusion criteria, age, primary cancer histology, paclitaxel administration schedule, and number enrolled in each arm) and interventions (pharmacological or non-pharmacological therapies, dose and administration routes of pharmacological agents), risk of bias, duration of follow-up, and primary outcome for all included studies.

For the primary outcome, the number of patients in the treatment and control arms who experienced PIPN and that of patients assessed at the endpoint was extracted to estimate a risk ratio (RR) and 95% confidence interval (CI). For studies that reported participant numbers at different chemotherapy cycles, the last data point was extracted. To avoid heterogeneity introduced by studies that used different CIPN evaluation questionnaires, the participant numbers across neuropathic grades were summed for studies that reported patient numbers stratified by grade of neuropathy.

#### Assessment of Risk of Bias

Two authors independently assessed risk of bias of included studies using Cochrane Collaboration’s risk of bias tool on the following domains: allocation sequence generation; allocation concealment; blinding of participants and personnel; blinding of outcome assessment; incomplete outcome data and selective reporting (6). Differences were resolved by discussion.

#### Assessment of Heterogeneity

Visual inspection of forest plots, chi-square tests, and the *I* ^2^ statistic assessed heterogeneity between studies. A *p*-value higher than 0.10 for the chi-square test and an *I* ^2^ value lower than 25% was interpreted as a low level of heterogeneity.

#### Data Synthesis

When sufficient clinically similar studies were available, their results were pooled in the meta-analysis. For the primary outcome, the RR for each study was calculated and combined using the random-effects model based on the Mantel-Haenzsel method (7). An RR of less than 1 indicated an advantage for the neuroprotective intervention.

#### Subgroup Analyses

Subgroup analyses determined *a priori* were performed for routes of administration of pharmacological agents (oral versus parenteral). Additional subgroup analyses were conducted including studies deemed to have clinically similar interventions. Any statistically significant differences in primary outcome measure between subgroups was determined by testing for heterogeneity.

#### Quality of Summarized Evidence

We determined the quality of evidence using Grading of Recommendations Assessment, Development and Evaluation (GRADE) criteria for the following domains: risk of bias of included studies; inconsistency; indirectness, imprecision, and publication bias.

## Results

### Search Strategy Results

**Figure 1:**
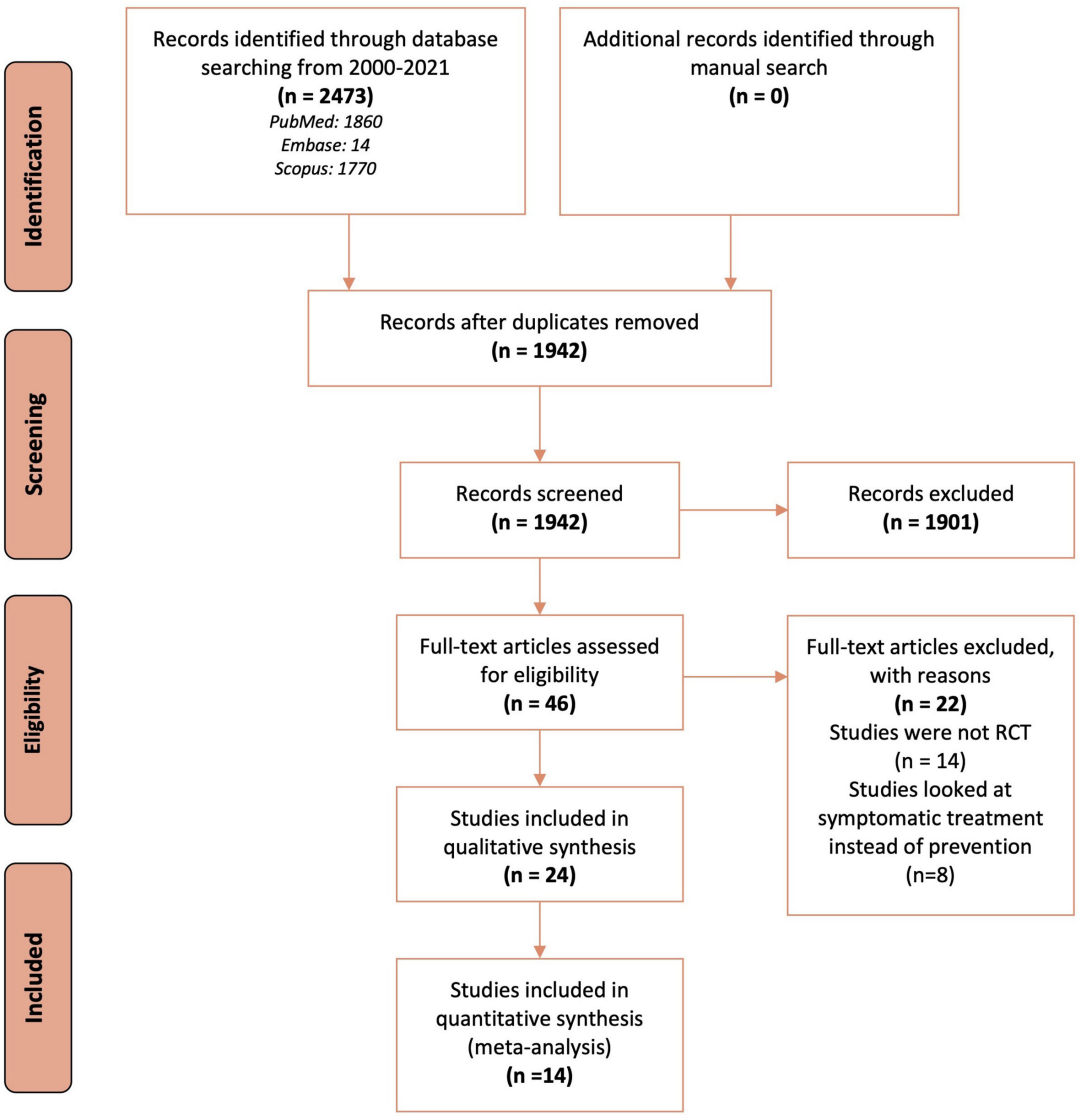
Flow chart describing the search and selection of studies according to the PRISMA guidelines.

Database search identified 2,473 records, of which following de-duplication and screening, 46 full-text articles were retrieved for further assessment and 24 RCTs met the inclusion criteria (**Figure 1**). Of these, 14 (seven pharmacological and seven non-pharmacological) were included in quantitative synthesis (meta-analysis).

To evaluate the presence of neuropathy, 11 studies used validated questionnaires based on self-reported symptoms (8–18). Seven studies used objective measures such as nerve conduction studies, electrophysiological studies, sensory testing, or balance scores (19–25). Six studies used a combination of questionnaires and objective measures (26–31). The studies primarily included breast cancer patients treated with paclitaxel.

### Characteristics of Included Studies

Of the 24 included studies, 12 investigated pharmacological agents while the other 12 investigated non-pharmacological interventions (**Tables 1** and **2**).

**Table 1:** Characteristics of included studies describing pharmacological interventions to reduce the incidence of paclitaxel-induced peripheral neuropathy.

Author	Year	Total sample size	Median age	Population statistics: Percentage of (%)		Intervention	Type of control	Primary outcomes	Outcome Measures
				Female patients	Patients with breast cancer				
Aghili (19)	2019	40	45.1	40 (100)	40 (100)	Gabapentin dose (intervention group) or placebo capsules, orally prescribed—300 mg for the 1st day, 600 mg for the 2nd day, and 900 mg divided into three doses for the 3rd up to the 14th day of each cycle.	Placebo	Incidence of both subjective and objective neuropathy.	1. Relative frequency of neuropathy (subjective) 2. Change in Nerve Conduction Velocity in electrophysiological nerve studies (objective)
Anoushirvani (20)	2018	63	51.5	46 (73)	36 (57)	Omega-3 capsules of 640 mg three times a day while receiving taxol, or vitamin E supplements at a dose 300 mg twice daily, or placebo capsules for the same period.	Placebo	To assess the efficacy of omega-3 and vitamin E on preventing PIPN *via* electrophysiological nerve studies and clinical evaluation.	1. Clinical and electrophysiological evaluation before the onset of chemotherapy and at months 1 and 3; presence of neuropathy and its progression was recorded by the neurologist
Argyriou (27)	2006	37	57	23 (62.2)	19 (51.4)	Oral supplementation of synthetic DL-alfa-tocopheryl acetate (Eviol soft gelatin capsules) at a dose of 300 mg/day twice daily during chemotherapy and up to 3 months after its suspension.	Standard of care	To estimate the efficacy of vitamin E supplementation in preventing PIPN by determining significant differences in PIPN occurrence between study arms.	1. Peripheral Neuropathy score 2. Neurological Symptom Score 2. Neurological Disability Score 2. Hughes’ Functional Grading Scale
Davis (22)	2005	117	58	53 (45.3)	Not reported	Study drug was given subcutaneous daily for 7 days starting the day before chemotherapy. Patients were randomized to receive low-dose rhuLIF (2 mg/kg), high-dose rhuLIF (4 mg/kg), or placebo.	Placebo	To observe a change in neurologic assessments, measured using standardized composite peripheral nerve electrophysiology score, from baseline to the end of cycle 4.	1. Standardized composite peripheral nerve electrophysiology score; based on nerve velocities and amplitudes 2. Vibration perception threshold 3. H-reflex latency 4. Symptom scores 5. Quantitative assessment of neurologic signs 6. NCI CTC 7. Production of rhuLIF antibodies (For toxicity)
Ghoreishi (21)	2012	69	45.9	69 (100)	69 (100)	Omega-3 fatty acid oral supplements as soft gelatin capsules at a dose of 640 mg three times a day during chemotherapy with paclitaxel and one month after the end of therapy or placebo of Sunflower soft gelatin capsules.	Placebo	To evaluate Reduced Total Neuropathy Score as a measure of the existence and severity of PIPN in patients.	1. Reduced Total Neuropathy Score (subjective sensory symptoms, pin sensibility, deep tendon reflexes, and nerve conduction studies of sural and peroneal nerves) 2. Nerve conduction study unilaterally using Nicolet/VIASYS Viking Quest electromyography Machine based on standard methods, Serum levels of omega-3 fatty acids - Motor conduction assessment: Distal motor latency, peak to baseline amplitude of compound muscle action potential and motor conduction velocity for tibial, peroneal, and ulnar nerves - Sensory nerve conduction of sural and ulnar nerves: peak-to-peak amplitude measurement of sensory action potentials and sensory conduction velocity (antidromic technique)
Hershman (12)	2018	409	53	409 (100)	409 (100)	Six active capsules, each containing 590 mg of Acetyl-L-carnitine hydrochloride (provides 500 mg of Acetyl-L-carnitine) and 10 mg of cellulose, were given to the intervention arm, while the control arm received six capsules containing 600 mg of cellulose.	Placebo	To determine the FACT-NTX score at each time point (1 and 2 years).	11-item FACT-NTX symptom module as a continuous measure (range = 0–44); a lowering in the FACT-NTX score (worse CIPN) of more than 10% or five points is considered clinically significant
Hilpert (11)	2005	72	Not reported	72 (100)	0 (0)	I.V. premedication with amifostine (thiophosphate cytoprotectant) 740 mg/m^2^ or placebo.	Placebo	To assess whether amifostine reduces chemotherapy-induced neurotoxicity of a first-line therapy with standard carboplatin/paclitaxel or with additional epirubicin in advanced ovarian carcinoma in comparison with a placebo treatment, as determined by the measurement of vibration perception thresholds and vibration disappearance thresholds before cycle 4 and after the end of treatment.	1. Vibration perception thresholds and Vibration disappearance thresholds before cycle 4 and after the end of treatment 2. Patella and Achilles tendon reflex activities 3. Two-point-discrimination [back of the hands, 10, 5, 3, and 1.5 cm separation; tibia (vertical), 10 and 4 cm separation] 4. Specific sensory symptoms and fine and global motor activities, surveyed *via* a patient questionnaire 5. EORTC QLQ-C30 questionnaire 6. Toxicity including sensory neuropathy and pain categories according to NCI-CTC
Khalefa (13)	2020	65	Not reported	65 (100)	65 (100)	For the intervention groups, the low-dose group received paclitaxel in addition to N−acetylcysteine 600 mg twice daily for 12 weeks while the high-dose group received N−acetylcysteine 1,200 mg twice daily for 12 weeks. The control group received paclitaxel only.	Standard of care	To determine the incidence of grade II or more PIPN at 12 weeks.	1. CTCAE v4.0, assessed weekly for 12 weeks 2. Modified Total Neuropathy Score at baseline and after 6 and 12 weeks 3. FACT/Gynecologic Oncology Group-NTX subscale assessed at baseline and after 6 and 12 weeks 4. Blood samples withdrawn from patients at baseline and after 12 weeks to evaluate serum levels of malondialdehyde and nerve growth factor using commercial spectrophotometric and ELISA kits, respectively
Leal (10)	2014	185	63	150 (81)	Not reported	Patients received glutathione 1.5 g/m^2^ or placebo (100 ml of 0.9% saline) intravenously over 15 min immediately before second dose of chemotherapy.	Placebo	To measure sensory chemotherapy-induced peripheral neuropathy as measured repeatedly by the sensory subscale of the EORTC QLQ-CIPN20 during the first 6 cycles of chemotherapy.	1. EORTC QLQ-CIPN20 1. FACT for Patients with Ovarian Cancer assessments 3. CTCAE v4.0
Loven (26)	2009	43	59	43 (100)	0 (0)	On the first day of chemotherapy, every participant received one batch containing eight bottles of either glutamate 500 mg or identical-looking placebo capsules: 6 bottles for 6 cycles of chemotherapy, and 2 bottles for supplementation when capsules of a previous bottle were not enough due to delay of chemotherapy.	Placebo	To achieve a 65% reduction in the rate of patients with CIPN in the Glutamate group as compared with Placebo group as defined either by the rate of patients with signs and symptoms corresponding to severity scores 2–3 or by the rate of appearance of impaired electrophysiological features.	1. Rate of patients with signs and symptoms corresponding to severity scores 2–3 2. Rate of appearance of impaired electrophysiological features 3. Standard sensory–motor nerve conduction study using a Dantec Keypoint or Nicolet Voyager Electromyography 4. Specially designed questionnaire regarding the presence of tingling, numbness, pain, and muscle weakness
						1 capsule 3x daily, either half an hour before or 2 h after a meal, starting on that day.			
						This supplementation treatment was continued throughout the period of 6 cycles of chemotherapy and until 3 weeks later.			
Pachman (8)	2017	45	54.9	45 (100)	45 (100)	200 mg of minocycline (x2 100 mg capsules) on Day 1 followed by 100 mg twice daily or matching placebos until the 12 weeks of chemotherapy were completed.	Placebo	Obtain pilot data regarding the possible effect of minocycline on the prevention of PIPN and Paclitaxel acute pain syndrome.	1. Daily average Area Under Curve pain score 1. Acute pain syndrome questionnaire daily during chemotherapy to measure Paclitaxel acute pain syndrome 1. EORTC QLQ-CIPN20 questionnaire
Shinde (9)	2016	46	53.7	46 (100)	46 (100)	Pregabalin 75 mg or placebo twice daily, starting on the first night of chemotherapy and continuing through the planned 12 weeks of chemotherapy. During the 13th week, the dose was decreased to once a day at bedtime, after which patients went off-study.	Placebo	To assess the effectiveness of pregabalin on the Paclitaxel acute pain syndrome *via* the maximum of the worst pain scores for the week following the first cycle of paclitaxel administration, as measured by a question on the daily post-paclitaxel questionnaire.	1. EORTC QLQ-CIPN20 questionnaire 2. Patient-reported acute pain syndrome questionnaire 3. Maximum of the worst acute pain scores 4. Adverse events per CTCAE criteria

CIPN, Chemotherapy-Induced Peripheral Neuropathy; NCI-CTC, National Cancer Institute Common Toxicity Criteria; CTCAE, National Cancer Institute Common Terminology Criteria for Adverse Events; ELISA, Enzyme-linked immunosorbent assay; EORTC QLQ, European Organization for Research and Treatment of Cancer Quality-of-Life Questionnaire; FACT-NTX, Neurotoxicity component of the Functional Assessment of Cancer Therapy-Taxane; PIPN, Paclitaxel-Induced Peripheral Neuropathy; rhuLIF, recombinant human leukemia inhibitory factor.

**Table 2:** Characteristics of included studies describing non-pharmacological interventions to reduce the incidence of paclitaxel-induced peripheral neuropathy.

Author	Year	Total sample size	Median age	Population statistics: Percentage of (%)		Intervention	Type of control	Primary outcomes	Outcome measures
				Female patients	Patients with breast cancer				
Beijers (14)	2020	180	60	102 (57)	62 (34)	Elasto-Gel hypothermia frozen gloves worn bilaterally 15 min before, during the 1- to 2-h infusion of chemotherapy up till 15 min after the treatment. Frozen gloves were put in a freezer (−20°C) for 3 h prior and are changed every 45 min.	Standard of care	To assess incidence of CIPN by comparing scores of patients on the EORTC QLQ-CIPN20 scale.	EORTC QLQ-CIPN20 scale Secondary endpoints: CIPN single items, quality of life, tolerance of frozen gloves usage and dose reduction due to CIPN
Griffiths (28)	2018	46	47.3	46 (100)	46 (100)	Participants served as their own paired control, with randomization of the cooled glove/sock to either the dominant or the non-dominant hand/foot, worn for 15 min prior to, during, and 15 min after completion of the paclitaxel infusion.	Internal control	To assess symptoms of neuropathic pain, pain severity, and sensory sensitivity—measured with the Neuropathic Pain Symptom Inventory, Brief Pain Inventory, and Quantitative Sensory Testing, respectively—for the intervention versus control extremity at the end of the paclitaxel treatments, respectively. (primary outcomes not specified)	Neuropathic Pain Symptom Inventory for symptoms of neuropathic pain Brief Pain Inventory for pain severity Quantitative sensory testing for sensory sensitivity
Hanai (29)	2018	45	56	45 (100)	45 (100)	Patients wore frozen gloves and socks on the dominant side for 90 min, including the entire duration of drug infusion.	Internal control	To assess the incidence of CIPN (any grade), defined as a decline in tactile sensation from the pre-treatment baseline, as assessed by the Semmes-Weinstein monofilament test, which is a validated measure of peripheral neuropathy.	Patient-reported subjective symptoms Tactile sensation assessed by the Semmes-Weinstein monofilament test Thermosensory disturbance Manipulative dexterity Vibration perception Electrophysiological signs Cryotherapy tolerability Pharmacokinetics
Izgu (31)	2018	40	45.8	40 (100)	40 (100)	Patients in the intervention group went to a special room with thermostatically controlled temperature (20–22°C) to receive classical massage sessions 1 h before paclitaxel infusion for 30 min during days of chemotherapy once a week.	Standard of care	To assess for the presence of peripheral neuropathic pain and CIPN-related quality of life assessed by the Self-Leeds assessment of neuropathic symptoms and signs and EORTC QLQ-CIPN20 and with nerve conduction studies as an objective outcome.	Self-Leeds assessment of neuropathic symptoms and signs and EORTC QLQ-CIPN20—assessed at baseline and weeks 4, 8, 12, and 16. Nerve conduction study findings—baseline and week 12
Kanbayashi (15)	2020	38	57.6	38 (100)	38 (100)	On one hand (intervention), patients wore frozen flexible gloves (Elasto-gel or Cool Mitten) from 15 min before start of infusion of nab-PTX till 15 min after continuously. On the other hand (control), patients wore 2 surgical gloves (one size smaller than best fit) from 30 min before the infusion till 30 min after.	Internal control	To compare frequencies of CTCAE v4.0 grade ≥ 2 and Patient Neurotoxicity Questionnaire grade ≥ D (neuropathy interfering with Activities of Daily Living) peripheral neuropathies at the last evaluation between frozen glove-protected and surgical glove-protected hand.	Patient Neurotoxicity Questionnaire FACT-Taxane subscale Temperatures at each fingertip in both groups measured thermographically CTCAE v4.0
Kotani (18)	2021	49	52.5	49 (100)	49 (100)	Each patient donned two surgical gloves on each hand at every Paclitaxel infusion. Two one-size-smaller gloves were donned on one hand (study side) and two normal-size gloves were donned on the other hand (control side) over the 90 min from 30 min before the infusion to 30 min after the end of the infusion.	Internal control	To determine the difference in the incidence of Grade ≥ 2 CIPN (motor/sensory) between the study and control side.	Physician-reported CTCAE v4.0
Ng (30)	2020	38	55	38 (100)	38 (100)	The intervention group received cryotherapy *via* Elasto-Gel™ hypothermia frozen socks and gloves on all extremities from 15 min before paclitaxel until 15 min post-infusion every cycle.	Standard of care	To determine proportion of participants reporting Grade C-E symptoms on Patient Neurotoxicity Questionnaire at 2 weeks after 12 cycles of weekly paclitaxel.	Patient-Reported Outcomes questionnaires administered at baseline prior to paclitaxel, 1–2 weeks, and 3, 6, and 9 months post-paclitaxel treatment: Patient
									Neurotoxicity Questionnaire, EORTC QLQ-CIPN20 Electrophysiological assessments conducted at baseline, 1–2 weeks, and 6 months post-paclitaxel treatment: Nerve Conduction Studies and Sympathetic Skin Response
Ruddy (16)	2019	42	54	41 (97.6)	42 (100)	On hands, patients wore cotton gloves inserted into the pocket of a quart-sized plastic bag 2/3 full of ice and another similar quart-sized plastic bag was placed on top of both hands if there were no IV lines. On feet, patients wore light cotton socks, which were then placed on top and beneath similar gallon-sized plastic bags half full of ice.	Standard of care	To assess the EORTC QLQ-CIPN20 score and measure Area Under Curve of EORTC QLQ-CIPN20 sensory scores over the 12 weeks of paclitaxel, adjusted for baseline scores.	EORTC QLQ-CIPN20 was completed at baseline, weekly ×12, then monthly ×6 Area Under Curve of EORTC QLQ-CIPN20 scores was calculated for subscale scores, adjusting for baseline, and compared between arms (Wilcoxon rank-sum test) CTCAE
Shigematsu (24)	2020	44	Not reported	44 (100)	44 (100)	Frozen (−20°C) gloves/socks (Elasto-Gel, mittens, and slippers) worn on both hands and feet continuously for 15 min before paclitaxel infusion until 15 min after infusion in accordance with previous report regarding cryotherapy (total 90 min) for each cycle.	Standard of care	To assess the percentage of patients with a significant decrease in their FACT-NTX score.	Patient Neurotoxicity Questionnaire CTCAE FACT-NTX score
Sundar (23)	2017	69	53	69 (100)	69 (100)	Continuous-flow limb hypothermia at a coolant temperature of 22°C using thermoregulator device supplying coolant (water) to limb wraps.	Internal control	To observe differences in nerve conduction studies carried out at baseline, 1 month into treatment, the end of treatment, and 3 months post-treatment.	Nerve conduction studies Total Neuropathy Score (safety and tolerance measures) Visual analog pain scale Subjective tolerance scale Shivering Assessment Scale
						Randomization for limb cooling was carried out and the non-cooled limb served as internal control prior to the first cycle of therapy, and the same limb underwent cooling for all subsequent cycles, while the non-cooled limb remained as control.			
Tsuyuki (17)	2016	43	60	43 (100)	43 (100)	For every nab-PTX infusion, each patient wore 2 surgical gloves (provided at room temperature) of the same size on their dominant hand for only 90 min: during the 30 min before the administration of nab-PTX, during the 30 min nab-PTX infusion itself, and during the 30 min after the end of the infusion.	Internal control	To assess the incidence of nab-PTX-induced peripheral neuropathy grade 2 or higher between the compression surgical gloves-protected hands and control hands using the CTCAE v4.0.	CTCAE v4.0 Patient Neurotoxicity Questionnaire Temperature changes of the tip of each finger using thermography
Vollmers (25)	2018	36	50	36 (100)	36 (100)	Following assessment by a sports scientist, patients were assigned to physical exercises on one hand. Intensity depended on age, weight and training state.	Internal Control	Primary endpoints were sway areas and Fullerton Advanced Balance Scale scores. Secondary endpoints were upper and lower extremity strength and the scores on subjective scales	Posturometry Chair Rising Test Fullerton Advanced Balance Scale EORTC QLQ-C30

CIPN, Chemotherapy-Induced Peripheral Neuropathy; CTCAE, National Cancer Institute Common Terminology Criteria for Adverse Events; EORTC QLQ, European Organization for Research and Treatment of Cancer Quality-of-Life Questionnaire; FACT-NTX, Neurotoxicity component of the Functional Assessment of Cancer Therapy-Taxane; nab-PTX, Nanoparticle albumin-bound paclitaxel.

For the 12 pharmacological RCTs, median sample size was 64. The age of included participants ranged from 30 to 74 years. Six RCTs included only female patients with breast cancer. The pharmacological interventions include gabapentin (19), omega-3 fatty acids (20, 21), vitamin E supplementation (alfa-tocopheryl acetate) (20, 27), minocycline (8), pregabalin (9), glutathione (10), glutamate (26), amifostine (11), recombinant human leukemia inhibitory factor (rhuLIF) (22), Acetyl-L-carnitine (12), and N-acetylcysteine (13).

For the 12 non-pharmacological controlled trials, median sample size was 43.5. The age of included participants ranged from 23 to 74 years. Nine trials included only female patients with breast cancer. The non-pharmacological interventions included cryotherapy (14–16, 23, 24, 28–30), classical massage (31), compression therapy (17, 18) and sensorimotor exercises (25).

### Risk of Bias of Included Studies

**Figure 2:**
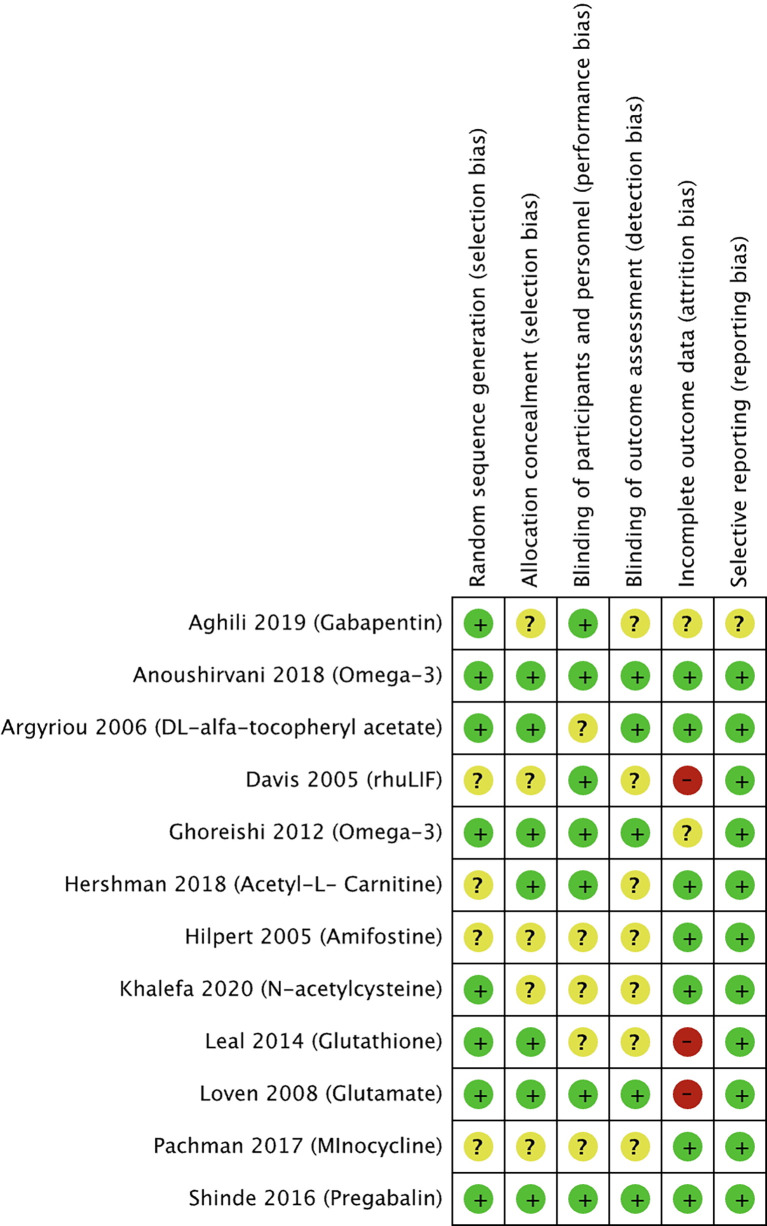
Risk of bias summary for studies investigating pharmacological approaches to prevent PIPN. Green circles represent low risk of bias, yellow circles represent unclear risk of bias, and red circles represent high risk of bias for their corresponding component categories.

**Figure 3:**
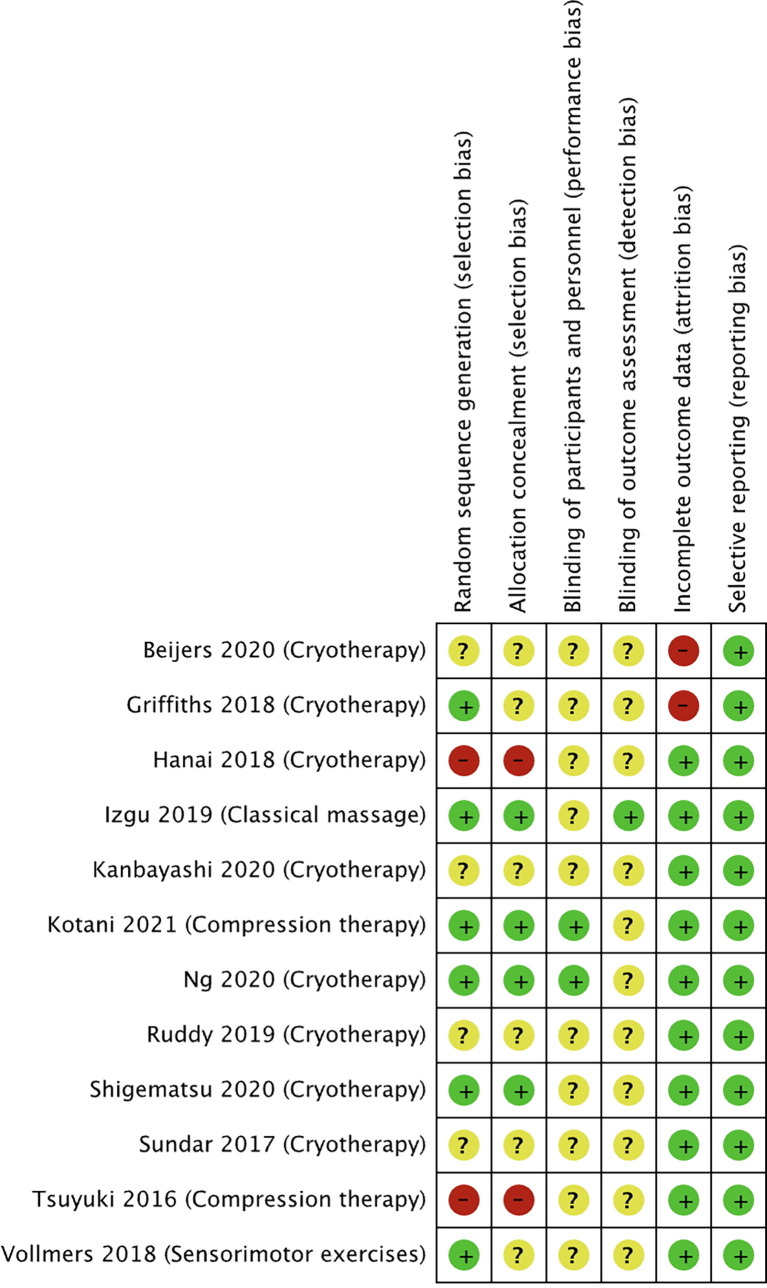
Risk of bias summary for studies investigating non-pharmacological approaches to prevent PIPN. Green circles represent low risk of bias, yellow circle represents unclear risk of bias, and red circle represents high risk of bias for their corresponding component categories.

The risk of bias for the selected studies is summarized in **Figures 2** and **3**.

Of the 12 selected pharmacological RCTs (**Figure 2**), four had unclear risk of selection bias as their methods for random sequence generation were not reported, while five had an unclear risk of selection bias with respect to the lack of reporting on allocation concealment. Blinding of participants and personnel was not reported in five trials, hence judged to have unclear risk of performance bias. Similarly, seven trials had an unclear risk of detection bias as blinding of outcome assessors was not reported. Two trials had an unclear risk, and three trials a high risk of attrition bias as more than 30% of the study participants were not included in the analysis for various reasons including loss of follow-up or disease progression. All trials were judged to have low risk of reporting bias, except one with an unclear risk.

Of the 12 non-pharmacological trials (**Figure 3**), two studies were at high risk of selection bias as the intervention was conducted on the participants’ dominant hands. Two studies were at high risk of selection bias as allocation was not concealed. All except one study had an unclear risk of performance bias as the blinding of the participants and study personnel was not reported. All except two studies had an unclear risk of detection bias the studies did not describe the blinding of outcome assessment. Two of the studies were at high risk of attrition bias as both studies had a 34% dropout rate, while all the other studies were at low risk. All the studies were at low risk of reporting bias.

### Pharmacological Interventions

12 controlled trials investigated the effect of pharmacological interventions on the prevention of neuropathy (**Table 1**).

#### Meta-Analysis

**Figure 4:**
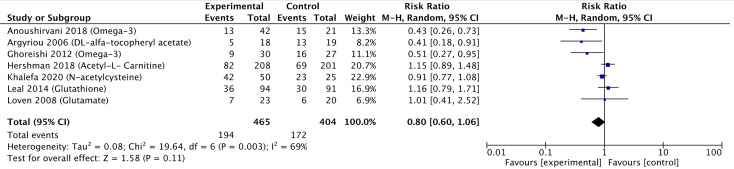
Forest plot comparing the incidence of PIPN between the experimental (pharmacological approaches) and control (placebo) arms. Black diamonds are the estimated pooled hazard ratio for each meta-analysis; blue box sizes reflect the relative weight apportioned to studies in the meta-analysis.

A meta-analysis including seven of the 12 pharmacological controlled trials was pursued (10, 12, 13, 20, 21, 26, 27) as they reported the number of participants corresponding to each grade of neuropathy, which allowed pooling of data. Three studies reported a significant reduction in the risk of neuropathy, while four studies did not find any statistically significant reduction. Pooling these estimates, we found a small reduction in the risk of peripheral neuropathy (RR 0.80, 95% CI 0.60–1.06) (**Figure 4**). The GRADE quality was judged to be low due to high risk of bias in the methodological components of the included studies and inconsistency and imprecision for the pooled results (**Table 3**). There was significant heterogeneity between individual trial results (chi-square *p* value = 0.003, *I* ^2^ = 69%). There were insufficient studies to pursue sub-group analysis stratified by intervention method (oral and parenteral).

**Table 3:** GRADE score on the quality of summarized evidence comparing the incidence of peripheral neuropathy between the experimental (pharmacological approaches) with control (placebo) arms.

Study design	Risk of bias	Inconsistency	Indirectness	Imprecision	Other considerations	GRADE score
Randomized trials	Serious	Serious	Not serious	Not serious	Publication bias strongly suspected	Low

#### Interventions That Reduced the Incidence of PIPN

The study by Aghili et al. (19), which investigated the effect of gabapentin, showed a statistically significant difference between intervention and placebo groups in terms of neuropathic grade based on the National Cancer Institute Common Terminology Criteria for Adverse Events version 4.0 questionnaire (CTCAE v4.0) and sensory nerve conduction. However, patient-reported symptoms varied across the different chemotherapy cycles. The study by Anoushirvani et al. (20) reported a significant difference between patients receiving Omega-3 or Vitamin E as compared to placebo, although no significant difference was seen in the electrophysiological variables measured and the study was limited by the small sample size (*n* = 21) per intervention. The study by Argyriou et al. (27) showed that patients who received Vitamin E were at lower risk of developing PIPN, although it is worth noting that the trial was limited by its small intervention sample size (*n* = 18) and lack of placebo. The study by Ghoreishi et al. (21) showed that patients who received Omega-3 fatty acid supplements were at lower risk of incidence of peripheral neuropathy, and a significantly higher (better) sural sensory action potential amplitude. It is worth noting, however, that the trial was limited by its relatively small sample size in the intervention group (*n* = 35). The study by Khalefa et al. (13) showed that patients who received N-acetylcysteine had a statistically significant reduction in the incidence of Grade 3 peripheral neuropathy. Similarly, however, the study was also limited by small intervention sample size (*n* = 42).

#### Interventions That Did Not Reduce the Incidence of PIPN

The study by Davis et al. (22), which investigated the effect of rhuLIF, showed that it was not effective in preventing, delaying, or diminishing CIPN caused by carboplatin and paclitaxel. The study by Hilpert et al. (11) showed that while Amifostine did show some protective effects in that a significantly delayed onset and accelerated recovery was observed, there were no differences in self-reported sensory or motor symptoms, which led the authors to conclude that Amifostine should not be routinely recommended. Leal et al. (10) showed that Glutathione did not have any statistically significant effect on the development of CIPN. Loven et al. (26) showed that long-term glutamate supplementation did not have any statistically significant effect on neurological examinations, questionnaires and nerve conduction studies. The study by Pachman et al. (8) showed that minocycline was able to reduce the pain score attributed to Paclitaxel acute pain syndrome, but no statistically significant difference was seen in CIPN scores as defined by European Organization for Research and Treatment of Cancer Quality-of-Life Questionnaire CIPN20 (EORTC QLQ-CIPN20) sensory scores. Similarly, Shinde et al. (9) showed that patients treated with pregabalin did not find a statistically significant difference in the pain score associated with Paclitaxel acute pain syndrome and CIPN as determined by EORTC QLQ-CIPN20.

#### Interventions That Increased the Incidence of PIPN

Only one study showed that patients were at higher risk of PIPN. Hershman et al. (12) showed that patients who were co-administered Acetyl-L-Carnitine during chemotherapy on average had worse peripheral neuropathy as determined by the neurotoxicity component of the Functional Assessment of Cancer Therapy-Taxane (FACT-NTX) symptom module. The CIPN also persisted throughout the two-year period following Acetyl-L-Carnitine discontinuation.

### Non-Pharmacological Interventions

12 controlled trials investigated the effect of non-pharmacological interventions on the prevention of neuropathy (14–18, 23–25, 28–31) (**Table 2**).

#### Meta-Analysis

**Figure 5:**
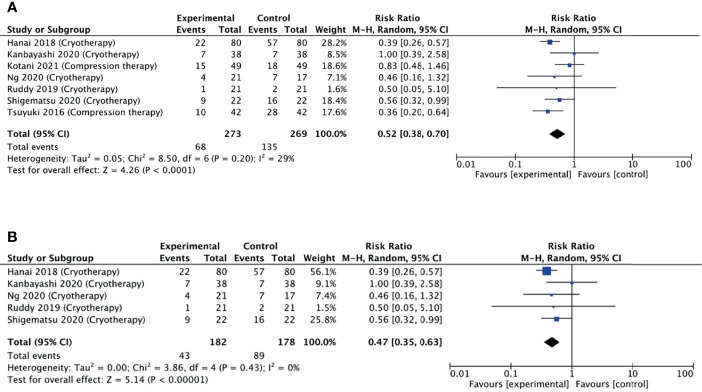
**(A)** Forest plot comparing the incidence of PIPN between the experimental (non-pharmacological approaches) with control (placebo) arms. **(B)** Forest plot comparing the incidence of PIPN between experimental (cryotherapy) and control (placebo) arms. Black diamonds are the estimated pooled hazard ratio for each meta-analysis; blue box sizes reflect the relative weight apportioned to studies in the meta-analysis.

A meta-analysis was pursued for studies that used non-pharmacological interventions, as per our protocol. Seven studies were included in the meta-analysis as they reported the number of participants corresponding to each grade of neuropathy. Six studies reported a reduced relative risk of neuropathy, while one study did not find any difference. Pooling these estimates, we found that non-pharmacological interventions were able to halve the risk of developing neuropathy (pooled RR = 0.52, 95% CI = 0.38–0.70, *p* < 0.001, *I* ^2^ = 29%) (**Figure 5A**).

The GRADE quality was judged to be moderate due to serious risk of bias in the methodological components of included studies but there were no serious inconsistencies between studies (**Table 4**).

**Table 4:** GRADE score on the quality of summarized evidence comparing the incidence of peripheral neuropathy between the experimental (non-pharmacological approaches) with control (placebo) arms.

Study design	Risk of bias	Inconsistency	Indirectness	Imprecision	Other considerations	GRADE score
Controlled trials	Not serious	Serious	Not serious	Not serious	NIL	Moderate

#### Cryotherapy

Limb cryotherapy was the most investigated intervention, with eight studies included in this analysis (14–16, 23, 24, 28–30). Six of these studies used frozen or cooled gloves to cool limbs (14, 15, 24, 28–30), one study used a thermoregulatory device (23), and one study involved immersing the patient’s cotton gloved hands into an ice bucket (16). Of these, one study did not find any difference in the incidence of neuropathy, while four studies found a reduced incidence (**Figure 5B**).

A subgroup meta-analysis including five of the eight studies was conducted. Pooling these estimates (**Figure 5B**), it was found that non-pharmacological interventions were able to significantly reduce the incidence of neuropathy (pooled RR = 0.47, 95% CI = 0.35–0.63, *p* < 0.001, *I* ^2^ = 0%). Additional sensitivity analysis also revealed that no single study had a substantial effect on the CI.

#### Compression Therapy

Two studies explored the effect of peripheral compression (through wearing smaller glove sizes) during chemotherapy infusion (17, 18). The study by Tsuyuki et al. (17) found that hands compressed using surgical gloves had significantly lower mean grade of neuropathy as compared to non-treated hands. Conversely, the study by Kotani et al. (18) found that surgical glove compression therapy was not effective in preventing the incidence of peripheral neuropathy. The limited number of studies precluded a meta-analysis.

Kanbayashi et al. (15) evaluated efficacy of compression therapy *via* surgical glove and cryotherapy *via* frozen gloves, and found that they showed comparable efficacy in preventing CIPN.

#### Other Therapies

Izgu et al. showed that classical massage therapy prior to chemotherapy infusion showed a significantly reduced relative incidence of neuropathy at week 12 of infusion, although the effect was not preserved at week 16 (31). Vollmers et al. showed that sensorimotor exercise during treatment period improved strength and balance, although the improvements were not statistically significant (25).

## Discussion

CIPN is a critical issue affecting delivery of chemotherapy and quality of life of survivors; hence, prevention is imperative. This meta-analysis assesses clinically explored neuroprotectants for reducing incidence of PIPN, aiming to interpret their therapeutic efficacy and identify promising directions. Importantly, this study includes newer non-pharmacological trials not included in previous similar reviews. This study evidences a relatively higher efficacy of non-pharmacological than pharmacological interventions in reducing the incidence of PIPN in the treatment arm compared to the control.

Overall, when comparing the forest plots, non-pharmacological interventions displayed a lower pooled RR of 0.52 compared to 0.80 for the pharmacological (**Figures 4** and **5**), supporting higher efficacy of non-pharmacological interventions in reducing PIPN incidence. Among the non-pharmacological interventions, there was a notable benefit observed by the subgroup of studies investigating cryotherapy with a pooled RR of 0.47. This suggests that cryotherapy, through its vasoconstrictive effects, may have the potential to reduce the incidence of peripheral neuropathy by limiting initial exposure damage during the chemotherapy infusion.

The inconsistency in study design across the pharmacological and non-pharmacological studies was substantial. Though the evaluated studies demonstrated low heterogeneity, a low GRADE score was observed when comparing the incidence of PIPN in the treatment to control arms for pharmacological interventions. However, a high GRADE score was observed for the non-pharmacological interventions (**Tables 3** and **4**). Placebo control was a commonly used standard in all pharmacological studies except one. In the non-pharmacological studies, most used internal controls, while one used a placebo to demonstrate efficacy. The non-possibility of relevant placebos in physical interventions is important to recognize and consider.

A variety of outcome measures was used to assess PIPN incidence and treatment efficacy in preventing PIPN. Of the included pharmacological studies, the most commonly reported outcome measure was nerve conduction studies, while the most commonly used outcome in non-pharmacological studies was the EORTC QLQ-CIPN20 questionnaire. Majority of the studies reported adverse events as per CTCAE criteria. The diversity of CIPN assessment methods is concerning, with implications in clinical trial design, result interpretation, and practice (3, 32). Several consortiums have been formed to address this and propose a standardized approach (33). This study further emphasizes the importance of a gold standard objective approach to assess and report CIPN outcomes.

Apart from the efficacy of the interventions in the prevention of neuropathy, it is also important to consider the limitations, in terms of their safety and tolerability. In this review, majority of studies did not observe a large dropout rate due to direct concerns of safety. However, in the field of cryotherapy, there have been reports of frozen gloves used for limb hypothermia being recalled over concerns of frostbite and patient intolerance (34). In the studies that investigated compression therapy, limitations such as the patient physique and amount of required compression were raised (18). Thus, further studies investigating the safety, tolerability, and reproducibility (across various population cohorts) of the proposed interventions are imperative.

### Study Limitations

While mitigated through a systematic search, there is a risk of sampling bias due to exclusion of gray or non-English literature. Studies that investigated combinations of antineoplastic drugs (which included paclitaxel) were not included. The included studies often reported the incidence of PIPN using varying assessment tools. This precluded further investigation of the effect of these interventions on a reduction in the severity of neuropathy. Furthermore, methodological heterogeneity was observed across studies and inconsistencies in reporting were observed, which limits the conclusion strength for pharmacological interventions.

### Practice Implications

The included neuroprotective interventions are promising approaches in development. Low-to-moderate quality evidence suggests that non-pharmacological interventions are more efficacious than pharmacological agents explored in this study in reducing the incidence of PIPN. Specifically, subgroup analysis has revealed that limb cryotherapy appears to hold promise as a potential intervention for the prevention of PIPN. However, the strength of evidence is weak due to the small number of evaluable studies with small sample size. Considering the diversity in clinical and methodological aspects, the results should be interpreted with caution, and no absolute or general recommendations can be made.

### Research Implications

Relatively few clinical studies have explored non-pharmacological interventions for PIPN prevention. Some emerging ones include combination therapies such as cryocompression, and behavioral interventions such as acupuncture and exercise therapy (35–38). Larger multi-center, well-designed trials should investigate the effects of these promising agents for more conclusive evidence. Future trials may also consider the combination of strategies—pharmacological, non-pharmacological, or both, in the prevention of peripheral neuropathy. Trials should use standardized objective assessment methods and well-defined primary and secondary outcomes to ensure validity. Experimental studies uncovering the mechanisms of action of these methods are required. These can advance treatment by incorporation of optimal therapeutic parameters.

## Conclusion

This meta-analysis assessed neuroprotectants for preventing PIPN and highlights the emergence of non-pharmacological interventions (especially cryotherapy and compression therapy). Evidence from the selected trials demonstrate a greater efficacy of non-pharmacological than pharmacological interventions in reducing the incidence of PIPN. The quality of evidence from the evaluated studies is overall low, and sample size is small. Meticulous planning of trial design and standardizing CIPN assessment techniques will greatly improve outcome reporting and ease judgement of prospective interventions for incorporation into clinical practice.

## Data Availability Statement

The original contributions presented in the study are included in the article/**Supplementary Material**. Further inquiries can be directed to the corresponding authors.

## Author Contributions

AL and DY contributed equally to the study. RS, YS, and AB conceptualized the research. AL, DY, CT, BT, and YS carried out the data curation, analysis, and interpretation. AL, DY, SF, and AB drafted the manuscript. RS, AM, HI, and YS reviewed the manuscript and provided critical feedback. All authors contributed to the article and approved the submitted version.

## Funding

This work was supported by the National Medical Research Council under its Clinician Scientist—Individual Research Grant (NMRC/CNIG/1167/2017); the National University Health System under its NUHS Summit Research Program—Cancer (NCSP N-171-000-493-001); and the National University of Singapore under its N.1 Institute for Health’s Translational Core.

## Conflict of Interest

RS has received honoraria from Bristol-Myers Squibb, Lilly, Roche, Taiho, Astra Zeneca, DKSH, and MSD; has advisory activity with Bristol-Myers Squibb, Merck, Eisai, Bayer, Taiho, Novartis, MSD, and AstraZeneca; received research funding from MSD and Paxman Coolers; and has received travel grants from AstraZeneca, Eisai, Roche, and Taiho Pharmaceutical. AB reports grants from Paxman Coolers Ltd., outside the submitted work.

The remaining authors declare that the research was conducted in the absence of any commercial or financial relationships that could be construed as a potential conflict of interest.

## Publisher’s Note

All claims expressed in this article are solely those of the authors and do not necessarily represent those of their affiliated organizations, or those of the publisher, the editors and the reviewers. Any product that may be evaluated in this article, or claim that may be made by its manufacturer, is not guaranteed or endorsed by the publisher.

## References

[1] Loprinzi CL . Prevention and Treatment of Chemotherapy-Induced Peripheral Neuropathy. In: UpToDate (2017). Available at: https://www.uptodate.com/contents/prevention-and-treatment-of-chemotherapy-induced-peripheral-neuropathy.

[2] Hershman DL Lacchetti C Dworkin RH Lavoie Smith EM Bleeker J Cavaletti G . Prevention and Management of Chemotherapy-Induced Peripheral Neuropathy in Survivors of Adult Cancers: American Society of Clinical Oncology Clinical Practice Guideline. J Clin Oncol (2014) 32(18):1941–67. doi: 10.1200/JCO.2013.54.0914 24733808

[3] Molassiotis A Cheng HL Lopez V Au JS Chan A Bandla A . Are We Mis-Estimating Chemotherapy-Induced Peripheral Neuropathy? Analysis of Assessment Methodologies From a Prospective, Multinational, Longitudinal Cohort Study of Patients Receiving Neurotoxic Chemotherapy. BMC Cancer (2019) 19(1):132. doi: 10.1186/s12885-019-5302-4 30736741 PMC6368751

[4] Argyriou AA Bruna J Marmiroli P Cavaletti G . Chemotherapy-Induced Peripheral Neurotoxicity (CIPN): An Update. Crit Rev Oncol Hematol (2012) 82(1):51–77. doi: 10.1016/j.critrevonc.2011.04.012 21908200

[5] Pike CT Birnbaum HG Muehlenbein CE Pohl GM Natale RB . Healthcare Costs and Workloss Burden of Patients With Chemotherapy-Associated Peripheral Neuropathy in Breast, Ovarian, Head and Neck, and Nonsmall Cell Lung Cancer. Chemother Res Pract (2012) 2012:913848. doi: 10.1155/2012/913848 22482054 PMC3312207

[6] Sterne JAC Savovic J Page MJ Elbers RG Blencowe NS Boutron I . RoB 2: A Revised Tool for Assessing Risk of Bias in Randomised Trials. Br Med J (2019) 366:4898. doi: 10.1136/bmj.l4898 31462531

[7] McDonald JH . Handbook of Biological Statistics. Baltimore, MD: sparky house publishing (2009).

[8] Pachman DR Dockter T Zekan PJ Fruth B Ruddy KJ Ta LE . A Pilot Study of Minocycline for the Prevention of Paclitaxel-Associated Neuropathy: ACCRU Study RU221408I. Support Care Cancer (2017) 25(11):3407–16. doi: 10.1007/s00520-017-3760-2 28551844

[9] Shinde SS Seisler D Soori G Atherton PJ Pachman DR Lafky J . Can Pregabalin Prevent Paclitaxel-Associated Neuropathy?–An ACCRU Pilot Trial. Support Care Cancer (2016) 24(2):547–53. doi: 10.1007/s00520-015-2807-5 26155765

[10] Leal AD Qin R Atherton PJ Haluska P Behrens RJ Tiber C . NCCTG N08CA (Alliance): The Use of Glutathione for Prevention of Paclitaxel/Carboplatin Induced Peripheral Neuropathy: A Phase III Randomized, Double-Blind Placebo-Controlled Study. Cancer (2014) 120(12):1890–7. doi: 10.1002/cncr.28654 PMC4047184 24619793

[11] Hilpert F Stahle A Tome O Burges A Rossner D Spathe K . Neuroprotection With Amifostine in the First-Line Treatment of Advanced Ovarian Cancer With Carboplatin/Paclitaxel-Based Chemotherapy–a Double-Blind, Placebo-Controlled, Randomized Phase II Study From the Arbeitsgemeinschaft Gynakologische Onkologoie (AGO) Ovarian Cancer Study Group. Support Care Cancer (2005) 13(10):797–805. doi: 10.1007/s00520-005-0782-y 16025262

[12] Hershman DL Unger JM Crew KD Till C Greenlee H Minasian LM . Two-Year Trends of Taxane-Induced Neuropathy in Women Enrolled in a Randomized Trial of Acetyl-L-Carnitine (SWOG S0715). J Natl Cancer Inst (2018) 110(6):669–76. doi: 10.1093/jnci/djx259 PMC6005110 29361042

[13] Khalefa HG Shawki MA Aboelhassan R El Wakeel LM . Evaluation of the Effect of N-Acetylcysteine on the Prevention and Amelioration of Paclitaxel-Induced Peripheral Neuropathy in Breast Cancer Patients: A Randomized Controlled Study. Breast Cancer Res Treat (2020) 183(1):117–25. doi: 10.1007/s10549-020-05762-8 32601973

[14] Beijers AJM Bonhof CS Mols F Ophorst J de Vos-Geelen J Jacobs EMG . Multicenter Randomized Controlled Trial to Evaluate the Efficacy and Tolerability of Frozen Gloves for the Prevention of Chemotherapy-Induced Peripheral Neuropathy. Ann Oncol (2020) 31(1):131–6. doi: 10.1016/j.annonc.2019.09.006 31912787

[15] Kanbayashi Y Sakaguchi K Ishikawa T Ouchi Y Nakatsukasa K Tabuchi Y . Comparison of the Efficacy of Cryotherapy and Compression Therapy for Preventing Nanoparticle Albumin-Bound Paclitaxel-Induced Peripheral Neuropathy: A Prospective Self-Controlled Trial. Breast (2020) 49:219–24. doi: 10.1016/j.breast.2019.12.011 PMC7375545 31901783

[16] Ruddy KJ Le-Rademacher J Lacouture ME Wilkinson M Onitilo AA Vander Woude AC . Randomized Controlled Trial of Cryotherapy to Prevent Paclitaxel-Induced Peripheral Neuropathy (RU221511I); an ACCRU Trial. Breast (2019) 48:89–97. doi: 10.1016/j.breast.2019.09.011 31590108 PMC7558814

[17] Tsuyuki S Senda N Kanng Y Yamaguchi A Yoshibayashi H Kikawa Y . Evaluation of the Effect of Compression Therapy Using Surgical Gloves on Nanoparticle Albumin-Bound Paclitaxel-Induced Peripheral Neuropathy: A Phase II Multicenter Study by the Kamigata Breast Cancer Study Group. Breast Cancer Res Treat (2016) 160(1):61–7. doi: 10.1007/s10549-016-3977-7 27620884

[18] Kotani H Terada M Mori M Horisawa N Sugino K Kataoka A . Compression Therapy Using Surgical Gloves Does Not Prevent Paclitaxel-Induced Peripheral Neuropathy: Results From a Double-Blind Phase 2 Trial. BMC Cancer (2021) 21(1):548. doi: 10.1186/s12885-021-08240-6 33985457 PMC8120772

[19] Aghili M Zare M Mousavi N Ghalehtaki R Sotoudeh S Kalaghchi B . Efficacy of Gabapentin for the Prevention of Paclitaxel Induced Peripheral Neuropathy: A Randomized Placebo Controlled Clinical Trial. Breast J (2019) 25(2):226–31. doi: 10.1111/tbj.13196 30773731

[20] Anoushirvani AA Poorsaadat L Aghabozorgi R Kasravi M . Comparison of the Effects of Omega 3 and Vitamin E on Palcitaxel-Induced Peripheral Neuropathy. Open Access Macedonian J Med Sci (2018) 62018:1857–61. doi: 10.3889/oamjms.2018.333 PMC6236056 30455762

[21] Ghoreishi Z Esfahani A Djazayeri A Djalali M Golestan B Ayromlou H . Omega-3 Fatty Acids are Protective Against Paclitaxel-Induced Peripheral Neuropathy: A Randomized Double-Blind Placebo Controlled Trial. BMC Cancer (2012) 12:355. doi: 10.1186/1471-2407-12-355 22894640 PMC3459710

[22] Davis ID Kiers L MacGregor L Quinn M Arezzo J Green M . A Randomized, Double-Blinded, Placebo-Controlled Phase II Trial of Recombinant Human Leukemia Inhibitory Factor (rhuLIF, Emfilermin, AM424) to Prevent Chemotherapy-Induced Peripheral Neuropathy. Clin Cancer Res (2005) 11(5):1890–8. doi: 10.1158/1078-0432.CCR-04-1655 15756015

[23] Sundar R Bandla A Tan SSH Liao LD Kumarakulasinghe NB Jeyasekharan AD . Limb Hypothermia for Preventing Paclitaxel-Induced Peripheral Neuropathy in Breast Cancer Patients: A Pilot Study. Front Oncol (2017) 6(JAN). doi: 10.3389/fonc.2016.00274 PMC5222823 28119855

[24] Shigematsu H Hirata T Nishina M Yasui D Ozaki S . Cryotherapy for the Prevention of Weekly Paclitaxel-Induced Peripheral Adverse Events in Breast Cancer Patients. Support Care Cancer (2020) 28(10):5005–11. doi: 10.1007/s00520-020-05345-9 PMC7447649 32036471

[25] Vollmers PL Mundhenke C Maass N Bauerschlag D Kratzenstein S Rocken C . Evaluation of the Effects of Sensorimotor Exercise on Physical and Psychological Parameters in Breast Cancer Patients Undergoing Neurotoxic Chemotherapy. J Cancer Res Clin Oncol (2018) 144(9):1785–92. doi: 10.1007/s00432-018-2686-5 PMC11813415 29943097

[26] Loven D Levavi H Sabach G Zart R Andras M Fishman A . Long-Term Glutamate Supplementation Failed to Protect Against Peripheral Neurotoxicity of Paclitaxel. Eur J Cancer Care (Engl) (2009) 18(1):78–83. doi: 10.1111/j.1365-2354.2008.00996.x 19473225

[27] Argyriou AA Chroni E Koutras A Iconomou G Papapetropoulos S Polychronopoulos P . Preventing Paclitaxel-Induced Peripheral Neuropathy: A Phase II Trial of Vitamin E Supplementation. J Pain Symptom Manage (2006) 32(3):237–44. doi: 10.1016/j.jpainsymman.2006.03.013 16939848

[28] Griffiths C Kwon N Beaumont JL Paice JA . Cold Therapy to Prevent Paclitaxel-Induced Peripheral Neuropathy. Support Care Cancer (2018) 26(10):3461–9. doi: 10.1007/s00520-018-4199-9 29681015

[29] Hanai A Ishiguro H Sozu T Tsuda M Yano I Nakagawa T . Effects of Cryotherapy on Objective and Subjective Symptoms of Paclitaxel-Induced Neuropathy: Prospective Self-Controlled Trial. J Natl Cancer Inst (2018) 110(2):141–8. doi: 10.1093/jnci/djx178 PMC6007752 29924336

[30] Ng DQ Tan CJ Soh BC Tan MML Loh SY Tan YE . Impact of Cryotherapy on Sensory, Motor, and Autonomic Neuropathy in Breast Cancer Patients Receiving Paclitaxel: A Randomized, Controlled Trial. Front Neurol (2020) 11. doi: 10.3389/fneur.2020.604688 PMC7793726 33424755

[31] Izgu N Metin ZG Karadas C Ozdemir L Váetin N Demirci U . Prevention of Chemotherapy-Induced Peripheral Neuropathy With Classical Massage in Breast Cancer Patients Receiving Paclitaxel: An Assessor-Blinded Randomized Controlled Trial. Eur J Oncol Nurs (2019) 40:36–43. doi: 10.1016/j.ejon.2019.03.002 31229205

[32] Yeo F Ng CC Loh KWJ Molassiotis A Cheng HL Au JSK . Minimal Clinically Important Difference of the EORTC QLQ-CIPN20 for Worsening Peripheral Neuropathy in Patients Receiving Neurotoxic Chemotherapy. Support Care Cancer (2019) 27(12):4753–62. doi: 10.1007/s00520-019-04771-8 30972646

[33] Alberti P Cavaletti G Cornblath DR . Toxic Neuropathies: Chemotherapy Induced Peripheral Neurotoxicity. Curr Opin Neurol (2019) 32(5):676–83. doi: 10.1097/WCO.0000000000000724 31306214

[34] Medical Device Recall Notice Hypothermia Caps, Mittens and Slippers [Press Release]. Available at: https://www.accessdata.fda.gov/scripts/cdrh/cfdocs/cfres/res.cfm?id=162228.

[35] Bao T Seidman AD Piulson L Vertosick E Chen X Vickers AJ . A Phase IIA Trial of Acupuncture to Reduce Chemotherapy-Induced Peripheral Neuropathy Severity During Neoadjuvant or Adjuvant Weekly Paclitaxel Chemotherapy in Breast Cancer Patients. Eur J Cancer (2018) 101:12–9. doi: 10.1016/j.ejca.2018.06.008 PMC6147260 30007894

[36] Molassiotis A Suen LKP Cheng HL Mok TSK Lee SCY Wang CH . A Randomized Assessor-Blinded Wait-List-Controlled Trial to Assess the Effectiveness of Acupuncture in the Management of Chemotherapy-Induced Peripheral Neuropathy. Integr Cancer Ther (2019) 182019:1–4. doi: 10.1177/1534735419836 PMC6434440 30905173

[37] Kleckner IR Kamen C Gewandter JS Mohile NA Heckler CE Culakova E . Effects of Exercise During Chemotherapy on Chemotherapy-Induced Peripheral Neuropathy: A Multicenter, Randomized Controlled Trial. Support Care Cancer (2018) 26(4):1019–28. doi: 10.1007/s00520-017-4013-0 PMC5823751 29243164

[38] Bandla A Tan S Kumarakulasinghe NB Huang Y Ang S Magarajah G . Safety and Tolerability of Cryocompression as a Method of Enhanced Limb Hypothermia to Reduce Taxane-Induced Peripheral Neuropathy. Support Care Cancer (2020) 28(8):3691–9. doi: 10.1007/s00520-019-05177-2 PMC7316694 31811482

