# Supplementary material for: A Systematic Review and Meta-Analysis of the Effectiveness of Neuroprotectants for Paclitaxel-Induced Peripheral Neuropathy
Source: Front Oncol. 2022 Jan 5;11:763229. doi: 10.3389/fonc.2021.763229 (PMC8766304; doi:10.3389/fonc.2021.763229)
Supplement: Supplementary file 1 [file DataSheet_1.docx]

Supplementary Table 1. **Search Information**

| **Date of search** | 18 Sept 2021 |
| --- | --- |
| **Databases** | Medline (Via PubMed), Embase, Scopus |
| **Search string** | Paclitaxel neuropathy |
| **Search string exploded** | (("paclitaxel"[MeSH Terms] OR "paclitaxel"[All Fields] OR "paclitaxel s"[All Fields] OR "paclitaxels"[All Fields]) AND ("neuropathies"[All Fields] OR "neuropathy"[All Fields])) AND (meta-analysis[Filter] OR systematicreview[Filter])  **Translations**  **paclitaxel:** "paclitaxel"[MeSH Terms] OR "paclitaxel"[All Fields] OR "paclitaxel's"[All Fields] OR "paclitaxels"[All Fields]  **neuropathy:** "neuropathies"[All Fields] OR "neuropathy"[All Fields] |
